# Flying into the hurricane: A case study of UAV use in damage assessment during the 2017 hurricanes in Texas and Florida

**DOI:** 10.1371/journal.pone.0227808

**Published:** 2020-02-05

**Authors:** Faine Greenwood, Erica L. Nelson, P. Gregg Greenough

**Affiliations:** 1 Signal Program, Harvard Humanitarian Initiative; Harvard University, Cambridge, Massachusetts, United States of America; 2 Harvard T.H. Chan School of Public Health, Harvard University, Boston, Massachusetts, United States of America; 3 Humanitarian Geoanalytics Program, Harvard Humanitarian Initiative, Harvard University, Cambridge, Massachusetts, United States of America; 4 Department of Emergency Medicine, Harvard School of Medicine, Brigham and Women’s Hospital, Boston, Massachusetts, United States of America; Northeastern University, UNITED STATES

## Abstract

Unmanned aerial vehicles (UAVs) or drones have been used by disaster relief organizations in the United States since 2005. However, their place in the disaster response ecosystem—the standardization, utility, ethical, and legal challenges of drone use—remains largely unstudied. This case series describes how UAVs were used by two teams of responders for damage assessment purposes during the 2017 southeastern US Hurricanes Harvey and Irma. Data streams ranged from social media, direct observation, participant-observation and semi-directed interviews. Qualitative analysis was performed for thematic content derived from field observation and from post-hoc interviews. Outcomes of the qualitative analysis emphasize the barriers to deploying drones in the disaster context, their tactical implementation, programmatic integration, and ethical and legal challenges. These observations lay the groundwork for both future research on the utilization of drones and the prudent and ethical implementation of programs that employ drones in post-disaster settings.

## Introduction

### Constructs and definitions

Unmanned aerial vehicles (UAVs) or small unmanned aircraft systems (sUAS) are used throughout the world for many different purposes, including agricultural monitoring, infrastructure inspection, media production, and academic research. This paper uses the abbreviation “UAV” and “drone”, the more common colloquial term in the United States, as interchangeable. This study limits the scope of our inquiry to civilian sUAS (as defined by Part 107 to Title 14 of the United States Code of Federal Regulations) [1] that are utilized for the explicit purpose of damage assessment during disaster response. Disaster response is herein defined as “the provision of assistance or intervention during or immediately after a disaster to meet the life preservation and basic subsistence needs of those people affected”[2].

### Drones in disaster response: a brief history

Drones have been used to evaluate the impact of natural disaster for decades. In the US, UAV use in disaster response dates back to 2005, when the Texas A&M University Center for Robot-Assisted Search and Rescue (CRASAR) at the University of South Florida used small fixed-wing and helicopter-style UAVs to search for Hurricane Katrina survivors in Mississippi [3]. Soon after, a Federal Aviation Administration (FAA) memorandum [4] clarified that small UAVs could only be operated for non-hobby purposes (including disaster response operations) in U.S. airspace with a certificate of authorization (COA), citing concerns about disaster-response UAVs impeding the movement of other manned aircraft in crowded airspace.[5]

These COAs proved difficult to obtain (and were not available to private sector entities at all), greatly limiting the legal use of UAVs for disaster response in the US between 2005 and 2016. [6] UAV use for disaster response during this period was largely restricted to the U.S. Military, NASA, and a small number of other government agencies. The Western States Fire Missions project, a collaboration between the FAA and several other government agencies, was one such example: the project used a long-range UAV equipped with a multispectral scanner to gather information on active wildfires in forest areas. [7] Likewise, for the response to the 2014 devastating Rim Fire in California, the California Army National Guard and the Air National Guard 163d Reconnaissance Wing partnered with state fire fighters to use a Predator UAV to monitor fire movement. An after report praised the UAV imagery for its usefulness and detail, but noted that the Predator UAV was a “military asset” and that there was no mechanism by which the federal National Interagency Fire Center could request one; all UAV requests during disasters had to be cleared through the Secretary of Defense.[8]

While a number of civilian organizations attempted to fly UAVs for disaster response during this time, these experimental efforts often encountered considerable legal barriers. In 2013, Colorado experienced devastating flooding. CLMax Engineering, a local company, collaborated with the Boulder County Emergency Operations Center to collect imagery of the flooding with its company-owned UAV. After three days, the Federal Emergency Management Agency (FEMA) took over emergency operations, and subsequently ordered that the UAV be grounded. [9] The Texas Equusearch search and rescue (SAR) organization, which claims to have used UAVs for search and rescue operations since 2005, received a court order in February 2014 from the FAA ordering it to stop using drones for SAR activities.[10] In another incident in 2014, CRASAR intended to fly drones to assist in recovery after the Oso, WA mudslide; the organization ultimately was unable to fly after local authorities voiced privacy concerns.[11] In March of 2015, CRASAR collaborated with the Measure UAV consulting firm and the American Red Cross (ARC) to test UAV capacities in a post-disaster simulation with FAA approval. Their subsequent report on drone use for disaster response and relief operations described the technology as having “immediate benefits” for civilians communities and first responders, and called upon the FAA to make integrating UAVs into emergency and disaster response protocols a “top priority.” [6] I n particular, the report identified the lengthy COA process as an impediment to the swift deployment of UAV technology during disaster, observing that drones are “surprisingly underutilized” in disaster relief operations.

In 2016, the FAA issued the Small UAS Rule (14 CFR Part 107), which created a regulatory framework for the use of drones for commercial, non-hobby purposes. Part 107, as it is colloquially known, gave UAV users a clearer legal pathway towards UAV use as part of disaster response and assessment operations. This new regulatory environment, coupled with the decreasing price and increasing ease of use of civilian UAV systems, set the stage for the wider use of the technology in disaster response operations. In the spring of 2016, CRASAR also used UAVs to collect data during floods in Louisiana and Texas [12].

While the US imposed restrictions on the technology from 2005 to 2016, other nations with little or no restrictions continued to experiment with its use. A 2009 report from the United Nations Economic and Social Council on China’s response to the 2008 Wenchuan Earthquake noted that “unmanned micro aeroplanes equipped with remote sensors” were used by disaster responders to quickly collect information on the scope of the damage [13]. Responders to the 2011 Fukushimi Daiichi crisis in Japan used Honeywell T-Hawk micro air vehicles to safely inspect the damaged nuclear reactor, working in collaboration with experts from CRASAR. [14] I n 2012, UNOSAT, the United Nations satellite imagery analysis organization, used a UAV to deliver high-resolution imagery of Haitian camps for persons displaced by the 2010 earthquake to the International Organization for Migration (IOM) [15]. In 2013, the Royal Canadian Mounted Police located a missing man injured in a car crash with a Draganflyer X4-ES multirotor UAV, which had been equipped with an infrared sensor. The Draganfly company president speculated that the incident was the “first time that a life may have been saved with the use of a sUAS.”[16] Extensive media coverage of UAV use for mapping and disaster assessment during the response to Typhoon Haiyan in the Philippines in 2013 raised awareness of the technology’s potential uses amongst public and private voluntary disaster responders. [17,18] In 2014, the Humanitarian UAV Network was founded by a group of aid workers, with the explicit goal of providing an organizing body and sharing knowledge amongst individuals and organizations who wished to use the technology for disaster response purposes. Shortly afterwards, drones were used by disaster responders to evaluate structural damage in the aftermath of Cyclone Pam in Vanuatu in 2015; the earthquake in Nepal [19] later that year saw extensive UAV use by nongovernmental organizations (NGOs) for post-disaster mapping, [20] shelter mapping, [21] and search and rescue operations.[22]

As of 2018, numerous international aid and development organizations have launched programs focused on integrating small UAVs into disaster response and disaster assessment operations,[23] including the World Food Programme, [22] the UN High Commissioner for Refugees, [24] WeRobotics,[25]and the World Bank.[26] Organizations that use UAVs for disaster purposes cite their lower cost of collecting spatial data compared to other remote sensing platforms, their capacity to fly under cloud cover, and their ability to collect both video and extremely high resolution imagery. [27,28]

### Research questions and scope

Hurricanes Harvey and Irma struck the southern U.S. states of Texas and Florida in the late summer of 2017, two of the most destructive storms to make landfall in the continental United States since Hurricane Katrina in 2005. [29] The National Hurricane Center (NHC) attributed 68 deaths and $125 billion in damage to Hurricane Harvey, making it the second costliest storm in US history. [29] Hurricane Irma was ranked as the fifth costliest, inflicting an estimated $50 billion in damage and killing 129 people.[30]

While the adoption of UAV technology for use in such disasters is on the rise, there are a limited number of academic studies describing its prior use. CRASAR publications provide the most extensive known English-language body of work describing the real-world application of UAV technology in response to natural disasters.[31] However, these reports focus predominantly on the technological and tactical details of deploying UAVs for SAR or disaster assessment purposes. There remains a paucity of literature that addresses the subjective experiences of those attempting to integrate drone technology and drone-collected data into the greater disaster response ecosystem. This case study of these two disasters endeavors to address this gap through an ecosystem analysis and qualitative evaluation of actors deploying drone technology and those requesting and receiving UAV-captured, remotely-sensed products.

The study was reviewed and granted an exemption from the institutional review board of the Harvard T.H. Chan School of Public Health. Respondents were invited to review preliminary outcomes and this feedback was integrated into the final publication.

## Methods

### Design and data collection

Using a qualitative methodology, we explore how drones are being used for damage assessment after natural disasters, and what actors either deploying drones or using the data that they collect identify as key barriers to the technology’s wider adoption in damage assessment activities. Consolidated criteria for reporting qualitative research (COREQ) were applied, and can be viewed in S1 File. The study utilized four predetermined thematic domains of inquiry, including 1) tactical and technological challenges, 2) operational challenges, 3) ethical considerations, and 4) regulatory considerations. Thirteen narrative themes were then identified within these four domains of inquiry, which were used to initiate further discussion.

We used the case study methodology described by Robert K. Yin, in conjunction with other qualitative analysis methods.[32] Research was restricted to the “bounded system” of UAV use for disaster damage assessment in September 2017, the immediate aftermaths of Hurricanes Harvey and Irma. The qualitative analytical methodology used here follows that described by Green et al. [33] and Auerbach and Silverstein, [34] and incorporates multiple data streams, including semi-directed interviews, direct observation, participant-observation and the mining of academic literature, news articles and social media posts. Interviews and field notes derived from direct observation functioned as the primary sources of data, which were supplemented with data derived from social media postings and news media sources. Field notes, captured through digital recordings and note-taking, contained physical description of the environment in which UAV disaster responders were operating, immediate observations on the organizational and structural phenomena that witnessed, relevant technical details related to UAV hardware and software, and names and contact information of individuals in the field. These notes were shared between all researchers and utilized to “enhance data and provide rich context for analysis,” in alignment with best practices in qualitative research. [35] Text-files were uploaded to Dedoose and used as reference material during the coding process. All field subjects were informed of note-taking and consented to our collection of this information.

We used a shared Google Sheets-based data extraction tool, S3 File, to collect and quantify relevant social media postings, news articles, and other non-academic literature related to UAV use during Hurricanes Harvey and Irma. The majority of this collection took place immediately after Hurricane Harvey made landfall. Using Twitter and Facebook, we ran searches multiple times a day for items related to these search terms: drone, UAS, UAV, disaster response, search and rescue, and SAR. These searches used Twitter and Facebook’s internal search tools, and were conducted in compliance with Twitter and Facebook terms of service. Items directly related to UAV use by disaster responders were included in the resulting data extraction tool, and categorized by source, media platform, type of information, known UAV pilots or users, and flight purpose, among other criteria. Relevant resources collected in these tools were reviewed and relied upon as key references as we conducted our ecosystem analysis of both Harvey and Irma.

Post-Hurricane Harvey, the research team was embedded with a newly-developed, non-governmental UAV disaster response team in Rockport and in Houston, Texas. In Naples, Florida after Hurricane Irma, the team observed an academic partnership conducting UAV response. Both teams were identified via prior professional connections within the UAV community. During these cases, direct observation was conducted, focusing on both UAV users and those actors with whom they interacted. The research team did not participate in any of these actions ourselves; all field-level research was purely observational.

Following field observations, researchers conducted semi-directed telephone interviews utilizing an interview guide, S2 File, with the stakeholders previously observed, as well as with five other individuals who participated in UAV disaster response during Hurricanes Harvey and Irma, who were identified through respondent networks. These respondents included officials from non-governmental organizations, local government and fire departments, FEMA, and academic UAV programs all formally engaged in emergency operations. Interviewees were consented, then asked a series of questions pertaining to their personal experiences prior to and during the hurricane, with subsequent questions in the four domains of technical, programmatic, ethical and regulatory observations, and challenges of utilizing UAVs in post-disaster response. Each interview lasted for approximately 60 minutes. No other individuals beyond the respondents and the interviewer was present during these telephone calls. All interviews were recorded between September 2017 and March 2018, and then transcribed. The transcripts were not returned to the interviewees for comment, although interviewees were given an opportunity to review and comment upon a draft version of this manuscript.

For the two units of analysis—Hurricane Harvey in Texas and Hurricane Irma in Florida—the four domains of inquiry reached thematic saturation and generated no new alternatives or elaborations of the theory at approximately ten interviews. As saturation is more relevant than quantity for theoretical sampling in exploratory case studies, further interviews were deemed unnecessary. The ten key informants reviewed the case to provide construct validity and to maintain a qualitative chain of evidence.

### Data analysis

Interview transcripts were coded in each of the four domains of inquiry by the two primary authors using Dedoose qualitative research software, with blind coding employed to reduce researcher bias. Codes were initially developed a priori in consideration of themes derived from observational data with categories generated inductively. After the first round of coding, the lists and parent codes were refined based upon researcher agreement regarding key themes, semantics and thematic hierarchy. Each transcript was coded twice, first to establish broad impressions and then to develop and characterize relationships between thematic outcomes and the field context. These data were triangulated against observational data and the supplementary data garnered from academic and social media sources.

## Results

### Ecosystem traits and actors

The ecosystems of actors deploying UAVs and utilizing UAV-captured data for disaster response after Hurricanes Harvey and Irma were complex and differed in many ways. Furthermore, the tasking of UAV missions and UAV-captured data flows were nonstandard and often opaque. However, there are generalizable ecosystem traits that are summarized below.

Actors identified within the post-disaster UAV ecosystem were categorized into 1) primary deployers of UAVs, 2) primary utilizers of UAV-captured data, and 3) the actors that both deployed drones and utilized self-captured data. Primary deployers of UAVs can be further divided into five categories: i) unaffiliated individuals, ii) journalists, iii) actors providing volunteer services, iv) actors from governmental organizations, and v) private companies. Primary utilizers of UAV-captured data can be subdivided into i) disaster-affected individuals, ii) media and media consumers, iii) disaster response NGOs without in-house UAV capacity, iv) governmental actors, and vi) private companies. Those actors that both deployed UAVs and utilized self-captured data were largely confined to private companies that undertook surveys for insurance, inspection or construction purposes, as well as the media. Few outliers that had in-house UAV flight capacity and engaged in disaster response efforts were identified. The composition of subcategories, actions and transactions will be described below.

After Hurricanes Harvey and Irma, a large number of unaffiliated actors, often members of the affected communities, deployed drones from their homes or vehicles. While there were no reliable mechanisms to quantify the utilization of UAVs by unaffiliated actors, respondents agreed that the number of unaffiliated UAV pilots that deployed after Hurricane Harvey was significantly larger than any previous domestic disaster. Many of these pilots were drone ‘hobbyists’ prior to the hurricane and utilized consumer UAVs to gain situational awareness and create imagery of affected communities that they then broadcast to the world on social media outlets. Drone-captured videos flooded social media sites and were frequently adopted and amplified by traditional media outlets. [36–38]

Scant data are available on 1) how many of these pilots had FAA authorization, 2) their overall awareness of FAA regulations on UAV deployment during the hurricanes, and 3) how many of these pilots knowingly defied flight restrictions to capture the imagery they shared on the internet. While respondents in our study voiced legitimate concerns about these unaffiliated flights potentially crowding airspace and endangering low-flying disaster response vehicles, they also acknowledged that these flights likely produced situational data that was then used in post-hoc, non-standardized ways for disaster assessment and SAR.

Journalists equipped with UAVs also traveled to Texas and Florida prior to landfall, and posted some of the earliest aerial imagery of the damage. These actors (most of whom held FAA licenses and other operational authorizations) continued to deploy UAVs for media coverage of the extent of the hurricanes’ impacts, as well as to document search and rescue, response and recovery efforts.

**Fig 1:**
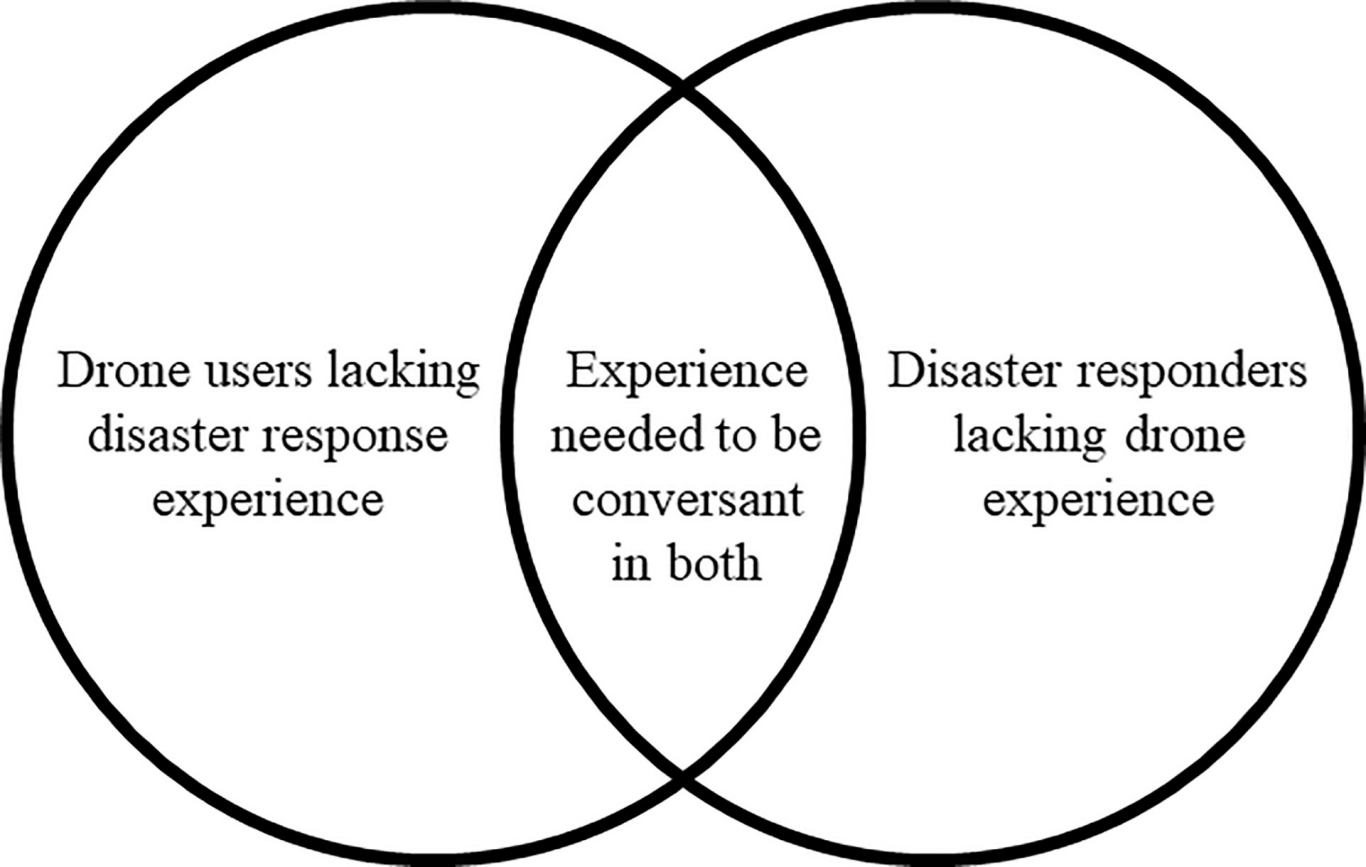
UAV Deployer’s Prior Experience. Venn Diagram depicting UAV deployers previous experience in post-hurricane response environments and drone utilization.

Actors observed in the post-disaster UAV ecosystem that provided volunteer UAV services included those affiliated with academic institutions, NGOs, private companies and unaffiliated volunteers. Of our respondents and observed actors, those operating under the auspices of the local government or an academic institution possessed experience utilizing UAVs in post-disaster environments, but prior disaster response experience was rare for actors associated with NGOs, private companies or those who were unaffiliated (Fig 1). These non-governmental, private or unaffiliated volunteers often had either UAV or disaster response professional backgrounds, but had scant experience utilizing drones for disaster response. All categories of volunteer UAV pilots employed both locals and non-locals, but there was a preponderance of volunteers from outside of the affected communities. Descriptions of UAV-produced products, tasking and data flows of these actors will be further described in the geographic-specific descriptions below.

Actors observed deploying UAVs under the auspices of a government entity represented diverse agencies, including FEMA, US Customs and Border Protection, and the Air National Guard [39]. Some government UAV deployers were individuals in local government organizations, such as police or fire departments. These actors were self-determined, often unilaterally deciding why, where and when to fly, as well as how to utilize footage. Study respondents who deployed UAVs for local government often operated as one-person teams and utilized the imagery products for 1) search and rescue, 2) situational awareness, 3) disaster assessment, and 4) to advocate for their communities with federal and state agencies.

Two compelling categories of UAV primary deployers were present after Hurricanes Harvey and Irma. The first was unaffiliated volunteers that deployed through network organizations that aggregate volunteers and imagery requests, task and coordinate missions, and facilitate data exchange. Two notable examples included the crowd-sourcing application DroneUp, LLC and the volunteer UAV network, Search with Aerial RC Multirotor (or SWARM).[40] The second was the partnership between a private entity, CyPhy Works, and the American Red Cross, through which a pilot project was launched to use a tethered drone for disaster assessment. [41]The tasking and data flow of these actors are further documented below.

### Regulatory environment

U.S. regulations regarding the deployment of UAVs are created and disseminated by the FAA; non-hobby operations, including disaster response flights, must adhere to the 2016 Small UAS Rule, 14 CFR part 107.[42] UAV operators for federal, state, and local government offices may apply to receive a COA, which provides either national or local permission to fly in any airspace class. [43] As of January 2020, there are no federal laws in place that specifically regulate drone data collection in the context of privacy protection.[44]

The FAA is authorized to issue Temporary Flights Restrictions (TFRs) that limit air travel (including small UAVs) due to temporary conditions, including disaster. Licensed UAV pilots who wish to fly in areas subject to a TFR are required to submit information in advance to the FAA’s System Operations Support Center (SOSC) via email. [45] In 2017, the FAA introduced an emergency waiver and authorization system known as the Special Governmental Interest (SGI) process, supporting UAV operations for the public good. Via this process, UAV operators may be issued expedited addendums to pre-existing COAs, or waivers and authorizations to Part 107 operators, enabling them to more quickly gain access to restricted flight areas.

During Hurricanes Harvey and Irma, TFRs were issued throughout disaster-affected communities in Aransas, Harris, Jefferson, Fort Bend and San Patricio counties in Texas and in Collier and Putnam counties in Florida. [46] The FAA disseminated information about these restrictions via TFR.FAA.gov and through the Twitter account @FAANews^.^ [47]Actors were encouraged to either contact the local airport or the local Flight Service Station at 1-800-WX-BRIEF for up to date information.

We observed that an actor’s knowledge of and adherence to these laws and restrictions was correlated with two factors: prior experience with non-hobby UAV flight, and prior familiarity with the U.S. disaster response and management system. Those actors operating under academic partnerships, from within the government, and who had worked with UAVs professionally in a non-disaster capacity had pre-existing experience with these structures, and frequently utilized a priori knowledge and collegial relationships to expedite authorizations and compliance. Unaffiliated volunteers and those working with NGOs appeared to be less aware, and were more frequently criticized by other UAV users for breaching laws and regulations.

### Texas

**Fig 2:**
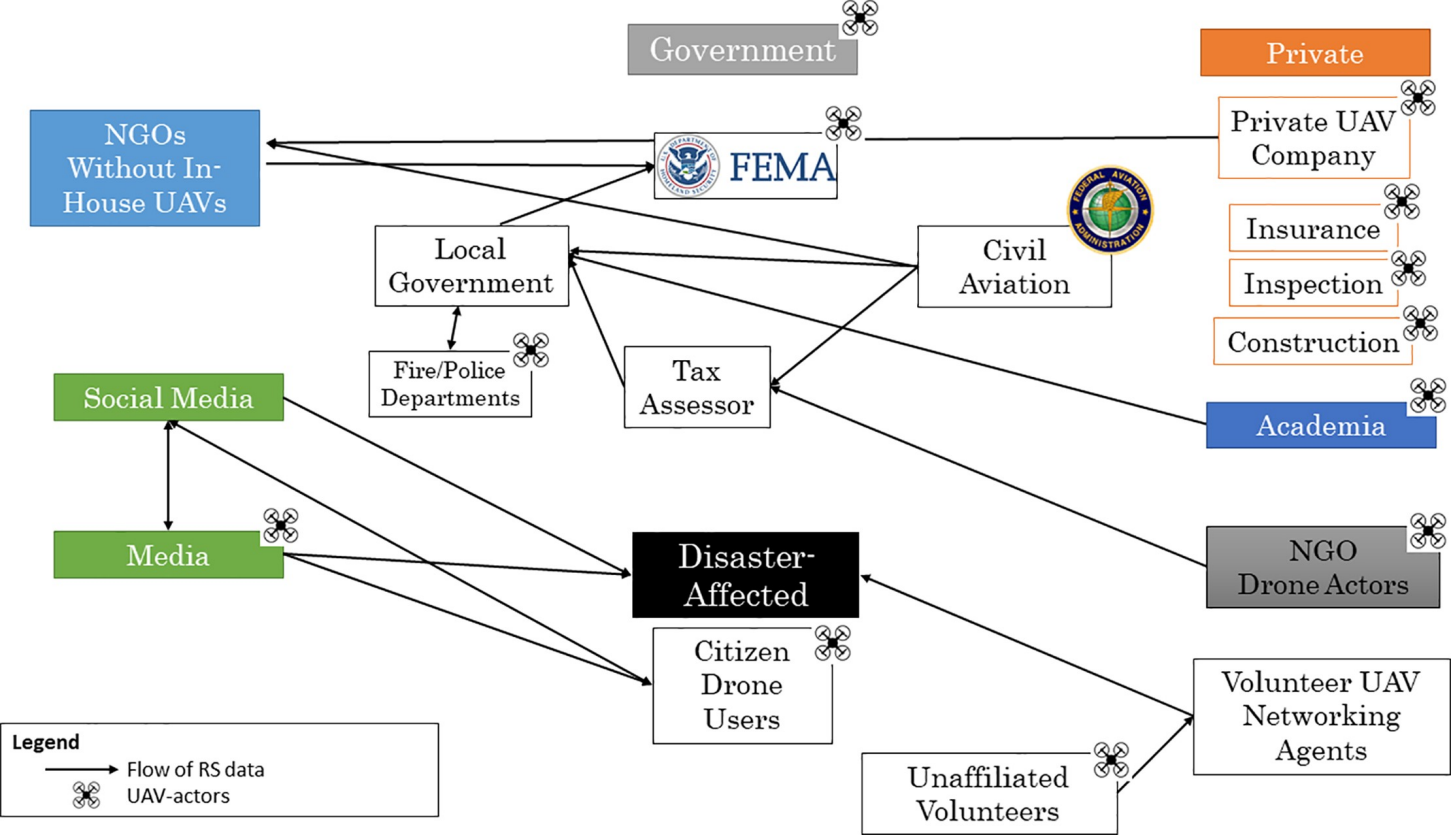
Ecosystem mapping of UAV-actors and utilizers of UAV-captured, remotely-sensed products after Hurricane Harvey, Texas, 2017.

The post-Hurricane Harvey ecosystem of civilian UAV use, including actors and data flows, is illustrated in Fig 2, below. All primary deployers, primary utilizers and actors that both deployed drones and utilized self-captured data were represented.

Within the governmental infrastructure, civil aviation provided remotely-sensed products acquired via manned aircraft to local governments and NGOs without in-house remote sensing capacity, such as the ARC. The California Task Force 3, one of FEMA’s 28 national response teams, and the North Texas UAV Response team, a collaboration between public safety agencies to support emergency response, deployed UAVs to assist in search and rescue efforts when canine and ground-vehicle efforts struggled with floods and debris. [48,49] The Texas State Guard Engineer Group for Hurricane Harvey used drones to identify hazardous material containers scattered by the hurricane. [50]Individuals within fire and police departments, such as the Houston Fire Department, deployed UAVs predominantly for situational awareness, but also for SAR and disaster assessment efforts. These data were shared within the government infrastructure and with FEMA to better target disaster response and recovery programs.

CRASAR, a center within Texas A&M University, led the largest civilian UAV deployment in any post-disaster response during Harvey.[51] Their collaboration with Florida State University, Kovar Associates LLC, and Lone Star UASC ultimately completed 119 flights,[52] mapping flood inundation and damage assessment. Thirteen pilots and two data managers were involved in operations, and at least one county official accompanied each UAV team during missions. Tasking was done by government officials, and data products were subsequently channeled back to those officials in the forms of video and maps.

The Humanitarian Drone Team (HDT), an NGO deploying UAVs for post-disaster response, originated in August 2017 as a direct response to Hurricane Harvey’s impending landfall. Founded by employees of private UAV companies, HDT was a direct product of social media conversations around the utility of drones in disaster response. The volunteer team was assembled and vetted through personal connections facilitated by Facebook. Resources were supplemented by a GoFundMe crowdfunding campaign, but otherwise were supplied by the volunteer team.

HDT’s deployment to Texas was compelled by an intended collaboration with a large national disaster response organization in Houston. Initially asked to map large areas of Houston and South Texas, the integration of HDT into disaster assessment efforts for this entity was ultimately unsuccessful. After contacting multiple local governments, the HDT relocated to the coastal community of Rockport, Texas to support the Aransas County Tax Assessors Office who tasked them for damage assessment mapping in Port Arthur, Holiday Beach and Rockport. Still images were geotagged and mapped using photogrammetry software, and videos were provided for situational awareness. These data were provided to the Tax Assessor and the Emergency Operations Center for integration into damage assessment reports for FEMA.

Network organizations such as DroneUp and SWARM provided mechanisms through which members of the affected community and response personnel requested imagery, and unaffiliated, volunteer UAV pilots identified missions. Both organizations acted as ports that channeled tasking and data between pilots and consumers. DroneUp is a limited liability corporation that created a Mission Match pilot management application. The Mission Match application receives requests from an affected community and directs volunteer UAV pilots through a UAS Traffic Management (UTM) platform to fulfill these missions. After Hurricane Harvey DroneUp claimed that more than 400 pilots signed up with the service, approximately one quarter of whom originated from the Houston area. [53] More than 5,000 missions using the platform were self-reported by DroneUp, ranging from checking on elderly neighbors and pets to infrastructure assessment for commercial operations.

Weeks after the landfall of Hurricane Harvey, ARC deployed a tethered drone for disaster assessment. This was an initiative funded by the UPS Foundation that aimed to determine the feasibility of deploying CyPhy’s Persistent Aerial Reconnaissance and Communications (PARC) platform in the post-hurricane environment. Tasking was provided by an ARC official that identified hurricane-damaged areas after analysis of previous surveys. Live feed video was analyzed in real time by a damage assessment specialist from the ARC, manually geotagged, and mapped onto another GIS platform. While rapid situational awareness was provided, respondents in our study noted that the tethered drone created overwhelming amounts of untagged video. Additionally, they described other significant challenges with this drone platform, ranging from access to launch-points to data management, analysis, and ultimately integration and utility of the data, itself.

The flow of UAV-captured data between all actors in this ecosystem, beyond the first recipient node, could not be mapped through discussion with respondents or through firsthand observation. Flow was unidirectional, in that remotely-sensed data was created by UAV operators and sent to utilizers without reciprocation, and often with minimal feedback from these utilizers. There was little to no sharing of UAV-captured data between UAV actors, and the degree with which UAV-captured data was shared between response organizations is uncertain. Similarly, UAV operators were uncertain of alternate drone actors operating in the space, or what was being mapped by other actors.

The lack of coordination, communication, or a data-sharing mechanism led to mapping redundancies, especially in Aransas and Harris counties. All respondents voiced concern regarding the lack of coordination and communication surrounding airspace use, data sharing, and tasking. These concerns are described in more detail in the thematic analysis section of this paper.

### Florida

**Fig 3:**
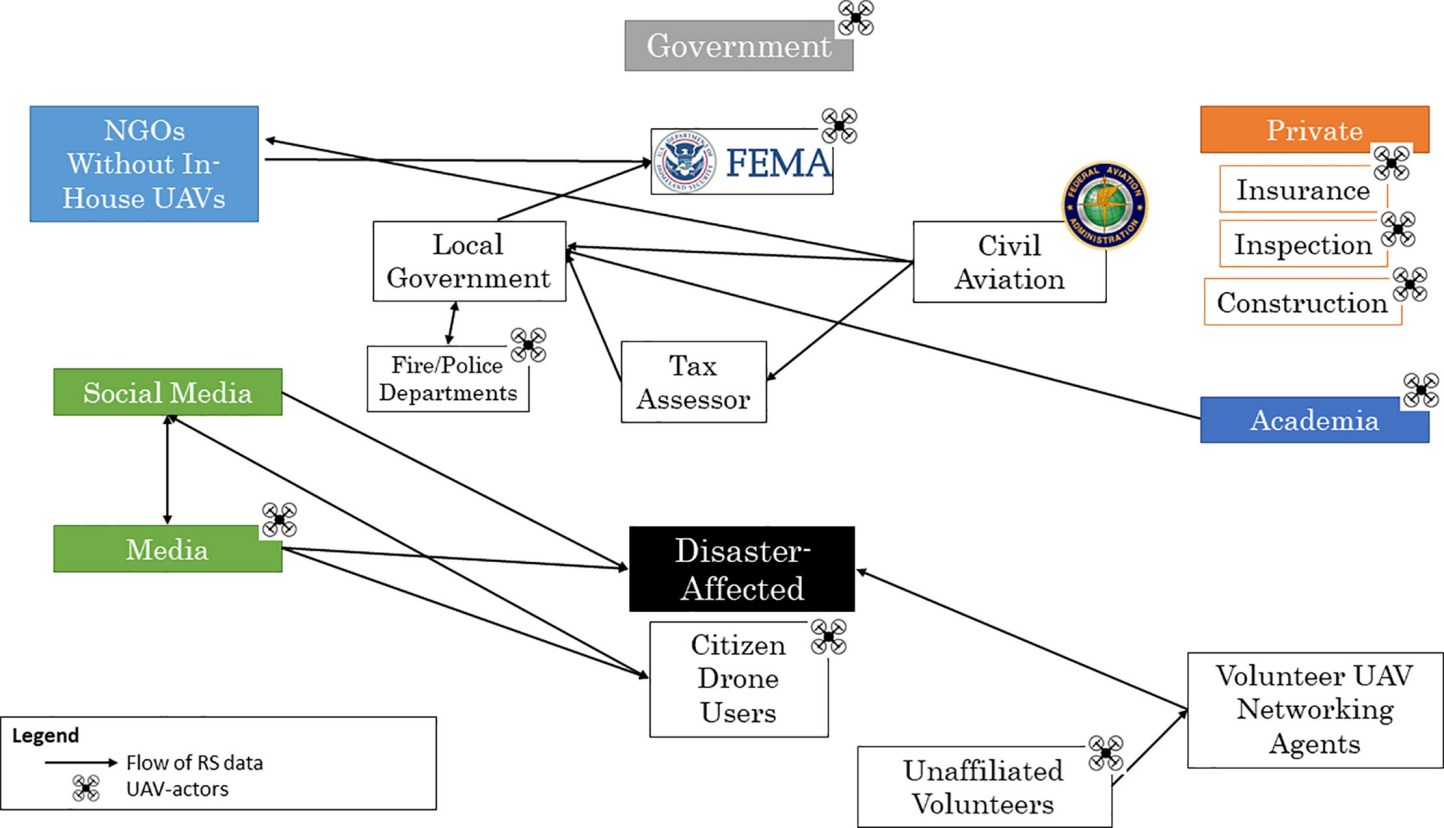
Ecosystem mapping of UAV-actors and utilizers of UAV-captured, remotely-sensed products after Hurricane Irma, Florida, 2017.

The utilization, tasking, and subsequent data flows from unmanned aerial vehicles after Hurricane Irma were broadly similar to those present in the post-Harvey ecosystem (Fig 3). Unaffiliated individuals from the community deployed drones for situational awareness and for sharing on social media. Media actors continued to produce, amplify and broadcast drone-captured imagery. Private entities utilized UAVs for inspection, insurance and construction purposes, and a few individuals from local governments deployed UAVs and incorporated those data into response efforts. Volunteer pilots tasked by DroneUp claimed that they surveyed over 300 square miles^.^ [54] SWARM deployed at least 12 pilots to assist in neighborhood damage assessments through the production of 3D maps.[55]

Again, the academically-affiliated volunteers deploying UAVs provided significant situational awareness to local authorities. Led by Florida State University’s Center for Disaster Risk Policy (CDRP) and in collaboration with CRASAR and Kovar LLC, volunteers deployed to Collier and Putnam counties, where they worked closely with the local Office of Emergency Management (OEM). Although involved in urban SAR oversight, this team was primarily tasked by the OEM to conduct FEMA property damage assessments.

Government officials identified and prioritized public assets for evaluation. These targets were parceled into missions to be flown by teams consisting of two pilots. Data products followed a standardized protocol created by CRASAR in collaboration with FEMA that included five picture stills (elevation views of all sides plus nadir) and overview videos. Orthomosaics were produced for larger targets. These data were provided to FEMA through an online platform and directly to the EOM geospatial personnel for analysis and utilization. Critical to these efforts were pre-existing relationships between all parties of the collaboration, as well as with disaster response and government officials. Familiarity with the post-disaster environment fostered an organized deployment with daily briefs and mission tasking, safety checks and communication mechanisms, data management protocols and debriefing. Volunteers were prepared for the post-hurricane context, brought the resources necessary to sustain the operation to avoid pulling resources from the affected community, and were knowledgeable of the regulatory environment. Overall, this team reported that they carried out a total of 247 flights and provided data on 491 critical infrastructure targets.

In general, there were significantly more actors deploying UAVs for post-disaster response in Texas after Hurricane Harvey than in Florida after Hurricane Irma. Non-governmental, volunteer UAV organizations were considerably fewer in the post-Irma ecosystem, although there were corporations that deployed drones for damage assessment without compensation.

While the etiology of this phenomenon is unclear, respondents suggested several variables that influenced volunteerism. Firstly, Houston is a large metropolitan center and was severely affected by prolonged rain and flooding. Florida’s urban centers were largely spared by Hurricane Irma, thereby reducing perceived impact and the need for volunteer involvement. “Disaster” or “compassion” fatigue, the principle that overexposure to disasters leads to a depletion of compassion or response, was also discussed, given how quickly Irma hit after Harvey.[56] Volunteer UAV pilots responding to Hurricane Harvey were doing so without monetary compensation, and likely with depleted monetary and psychological resources. Respondents cited not only monetary constraints that prevented deployment to Florida, but also frustration with organizational challenges that undermined integration into disaster assessment processes.

## Qualitative analysis

### Respondent characteristics

Demographics of the respondents are outlined in Table 1. The majority of respondents were male, but for anonymity purposes, exact gender identifiers are not provided. Sixty percent were primary deployers of UAVs, 20% were primary utilizers of drone-captured data, and 20% were actors that both deployed UAVs and utilized data. One interviewee had no prior experience with UAV technology before the 2017 hurricanes; two interviewees had begun to work with UAVs within the past year. The other interviewees’ prior experience with UAVs ranged from at least two years to at least six years.

**Table 1:** Respondent demographics.

Respondent	Affiliation
Respondent 1	NGO UAV Team
Respondent 2	NGO UAV Team
Respondent 3	Academically-Affiliated UAV Team
Respondent 4	Academically-Affiliated UAV Team
Respondent 5	Crowd-Sourcing UAV Platform
Respondent 6	Government Agency
Respondent 7	Fire Department UAV Team
Respondent 8	Fire Department UAV Team
Respondent 9	NGO Disaster Response Organization
Respondent 10	Private UAV Company

### Thematic outcomes

**Fig 4:**
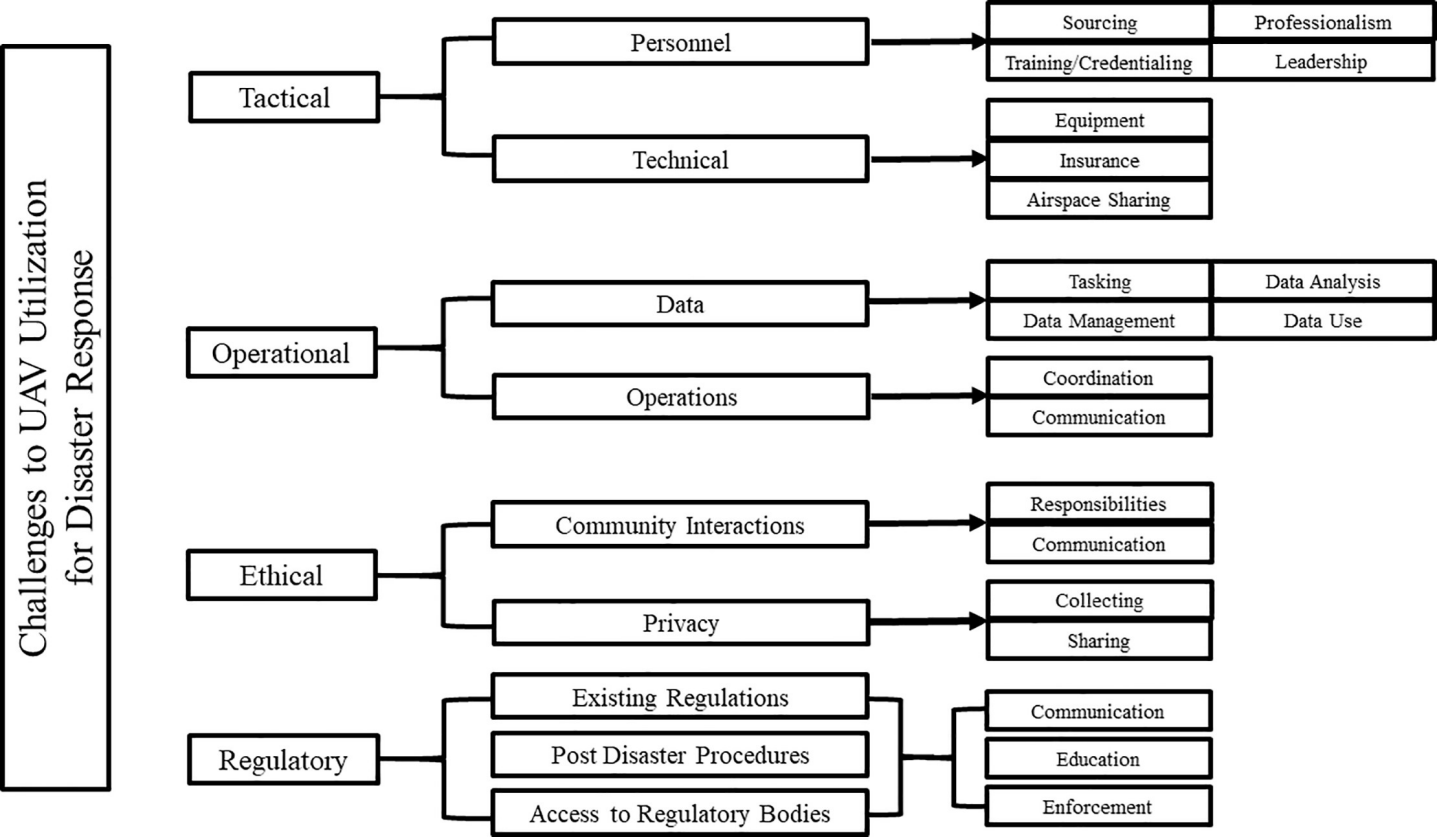
Coding tree identified through iterative qualitative analysis of interviews regarding the use of UAVs in disaster response.

After field observation, researchers identified four thematic domains of inquiry defined as: 1) tactical challenges, 2) operational challenges, 3) ethical considerations, and 4) regulatory considerations. Subcategories and twelve specific themes emerged during the coding process and were explored to saturation (Fig 4).

Fifteen thematic outcomes were identified in Table 2 as highly relevant to the discourse surrounding the use of UAVs for disaster response. These outcomes were categorized by the four domains of inquiry. Quotations are provided, but are not attributed to a specific respondent for the sake of anonymity.

**Table 2:** Thematic outcomes regarding the use of UAVs for disaster response.

Ethics	Regulatory	Operational	Technical
A lack of federal guidelines and standardized best practices regarding privacy protection forces drone uses to develop their own standards.	Most drone users were pleased with the FAA’s process of approving operations but some felt the agency did not understand the needs of disaster response drone teams.	Drone users do not find the FAA Part 107 credential adequate for assessing pilot skill in disaster response and would welcome a specialized system for training and evaluating volunteers.	While technical challenges were reported during the observational stage of this study, respondents did not identify either hardware or software factors as significant obstacles to their work.
Drone users are sympathetic to those with privacy concerns but are skeptical of poorly-informed objections.	Some drone users remained confused by the FAA approval system and found it to be an ‘unequal’ system in which those with pre-existing FAA contacts received faster responses.	Due to the lack of adequate evaluative mechanisms, drone pilots must rely upon personal relationships and experience to trust colleagues.	Drone pilots see themselves as data collectors, not data analysts or decision-makers in disaster response.
Interviewees reported largely positive interactions with the community and believed that the drone imagery provided clear benefits to community members.	Drone users wished the FAA and local law enforcement would enforce flight restrictions.	Respondents expressed frustration with poor communication, collaboration and data-sharing between disaster response organizations.	Interviewees are unclear regarding what happens to UAV-collected data and often lack feedback from end-users.
	Respondents expressed frustration with the bureaucracy imposed on their work and felt this ‘red tape’ might motivate some to operate outside of the system.	Drone users are distrustful of ‘disaster tourists’, differentiating themselves by emphasizing prior experience and the ability to be ‘self-sufficient’.	
		Drone users were motivated to prove that the technology is useful for specific disaster response purposes, but are still exploring optimal use cases.	

### Ethics

Ethics-related themes reflected interviewee concerns with ethical issues inherent in UAV use post-disaster. Predominantly, these identified themes centered around disaster responders’ interactions with members of communities affected by disaster and/or privacy considerations.

#### In the absence of federal guidelines regarding drone-collected data privacy, most drone users relied upon state and local regulations and internally-developed best practices for privacy protection and data sharing

Interviewees reported high general awareness of state and local laws pertaining to privacy and UAV data. While they were also aware of the ongoing legal controversy regarding the enforceability of these rules, they were not interested in challenging or defying local regulations. Interviewees did not cite specific best practices documents, such as the NTIA Voluntary Best Practices, but did describe internal and individual best practices pertaining to privacy protection.

A: “From an ethics standpoint, it's all going to come back down to that privacy concern again, and just making sure that those laws are respected … we're not capturing data that we don't need.”

B: “…We honor whatever rules, regulations, or laws are in place at that municipality, whether it be ordinances or state law. We're not gonna attempt to say anything different in regard to how people should behave with UAVs in those locations. I think it's important that we all respect everyone's privacy, whether it be looking in someone's window, or taking a drone and flying it across someone's backyard.”

#### Drone users are sympathetic to those with privacy concerns but are skeptical of what they consider to be poorly informed objections to drone use

Interviewees indicated that they were aware of and understood the basis of privacy concerns from the public. They indicated an understanding that they needed to engage in dialogue with community members around privacy issues. However, a majority of our interviewees were personally skeptical of commonly-expressed public concerns about drones and privacy. Some expressed concern that these arguments are rooted in a lack of knowledge about the technology and its capabilities, or about the current, regulatory environment.

A: “What stops you from walking down the street and somebody snapping a picture from across the street? It's not [any] different at the end of the day. I mean, I can look from my second story into my neighbor's backyard from my house. There is no expectation of privacy. That's a policy deal and [an] interpretation [of privacy laws].”

B: “From the civilian side, I get it. I don't want people flying over my kids, either. . . . I don't know, it's a very touchy question, and there's no solid answer to it. From my perspective, it's all about me going home every day, and it's all about my brother firefighters, sister firefighters, going home every day. The more information I have to get that higher situational awareness, the safer they are. Then again, from that civilian side, I think there just has to be a very open conversation, an open access to that information so that they understand why we need these things.”

#### Interviewees reported largely positive interactions with the community and believed that the drone imagery provided clear benefit to community members

The respondents reported that community members expressed gratitude for the data that they collected, particularly imagery depicting the post-hurricane condition of homes. One interviewee reported that they were “somewhat surprised” by this positive reception, echoing the commonly-held belief that drones are a widely distrusted technology. Another emphasized the importance of transparency as a means of “mitigating” negative responses from community members, noting that “most people, when you get to the show-them-what-you’re-doing kind of thing, are more impressed with it than anything else.”

A: “It was important because it provided some closure for our community… People in those communities could then see, “Do I still have a house? What is it that I need to do in order to prepare to get back into that house?”

B: “What we heard back, and what I've heard from people that lived down there that I know is that they were very appreciative of the [drone] data set. Because when you are evacuated out of your home and you can't get back into it, if you get somebody that can provide you with an overflight that shows if your house is flooded or it's not—it kind of puts ease to a person.”

### Regulatory

Themes related to regulation describe laws, rules, and the enforcement of those laws and rules by governmental organizations. This section also addresses interactions with regulatory bodies, such as the FAA.

#### Most drone users were pleased with the FAA’s process of approving flight operations in restricted areas, but some felt that the agency did not understand the needs of disaster response drone teams

The majority of interviewees expressed satisfaction with the FAA’s process of approving their flights in restricted areas. Still, they felt the system needed improvement. Interviewees pointed to a lack of FAA funding and a general sense that the FAA still failed to understand the specific needs of disaster responders. One interviewee described drone users’ interactions with the FAA as a “work in progress.”

A: “We had a very easy time dealing with them; they gave us priority attention; they spoke with us on the phone and helped us with any emailed requests for authorizations. Within hours every single time. We were never delayed by that other than an hour or two, but typically all of our delays happened because internally we didn't file the request that we should have.”

B: “I'm giving a lot of credit to the FAA. I think they're doing as much as they can for us with what they're able to do, but the government is unable to provide us with what we need right now.”

#### Some drone users remained confused by the FAA approval system and found it to be an “unequal” system in which those with pre-existing FAA contacts received faster responses

Some interviewees expressed concern about the inaccessibility of the FAA approval system, observing that it required pilots to have pre-knowledge of a specific contact and pre-existing personal relationships with the FAA to use it.

A “If you don't have that number, you're [‘out of luck’], so that system was frustrating. They needed a better way of allowing us to communicate our needs to them, but it needs to be open and above all, it needs to be fair. Everyone needs to have the same equal opportunity to get to that service.”

B: “Because we have preexisting relationships with the FAA, we were able to go in and within hours get permission to fly and so that made it real easy. If you don't have those connections, if the FAA doesn't know who you are—it is going to be a lot more difficult to try and get [permission].”

#### Drone users wished the FAA and local law enforcement would enforce flight restrictions

Some interviewees reported that they encountered unauthorized drone pilots in areas where they were authorized to fly. They noted that these pilots made it more difficult to operate. One interviewee reported that he told one of these drone pilots to leave the area, but the pilot refused, and local law enforcement declined to intercede in the matter when he asked them to do so. He and other interviewees found this lack of enforcement problematic.

A: “The FAA has shown, particularly in this past year, a complete unwillingness to enforce anything.”

B: “[The FAA’s] airspace system is pretty complete. It does everything that you need it to do to help manned pilots. Granted, there are a lot of us [drone pilots], and the majority of us are idiots … You know how you fix that? You enforce rules. You make sure that everybody is aware that the tools available to them are to be used responsibly and if you don't follow the rules, you're gonna get … taken out of the sky; you're not gonna be allowed to fly; you're not gonna be allowed to operate.”

#### Some drone users expressed frustration with the bureaucracy imposed on their work by the FAA and by other disaster responders and felt that this “red tape” might motivate some to operate outside of the system

A: “Treat us like we are … contributing members of the air space system, don't treat us like second class citizens because we're gonna end up just ignoring you and going around your guidance. Because if you're not gonna be participating in our process, why would we participate in yours? … If you're not gonna allow us access to the same tools that a manned pilot has for licensed pilots, why is that? You're issuing me a license—what good is that license if you're not gonna honor it with the things that I need done?”

B: “I have to do all of that in order to take a little piece of plastic and put it up 100 feet, maybe 50 feet in the air. That doesn't work for emergency response. . . . If you want me to respond to your emergency, you don't want me to have to go through all of that. But that's what government policy right now is stating that we have to do. “

### Operational

Operational themes pertain to the structure of and behavior of disaster-response organizations. In the context of this study, they concern the choices and resultant actions that humanitarian organizations take while they are preparing for and responding to a disaster.

#### Drone users do not find the FAA Part 107 credential adequate for assessing pilot skill, and welcome a more specialized system for training and evaluating volunteers

The majority of interviewees observed that the existing FAA Part 107 credential, a written test, was inadequate for assessing the tangible skills of drone pilots specifically in disaster response operations. They expressed a desire for a more detailed and specific mechanism for validating the skill of others and a standardized system for training drone pilots for disaster response.

A: “Part 107 was never designed for us. Part 107 was designed for commercial operations. It was never designed for public safety. As a volunteer or as a career disaster responder, it just kind of fills this really weird gap and doesn't do it well. We absolutely have to have something for everybody that speaks to operations for emergency response.”

B: “The biggest challenge we're seeing is delivering the training to the end-user and getting them to understand that because someone does something, just because you've got a piece of paper doesn't mean that you know how to deal with real-world scenarios or events.”

#### Drone users in disaster response must rely upon personal relationships and past experience to evaluate each others’ skill sets in the absence of standardized practical skill tests

Interviewees expressed the importance of trusting their collaborators. The vast majority of those interviewed had pre-existing personal relationships with the pilots with whom they worked. These were developed both via social media and via non-online interactions. Some interviewees directly connected this reliance on trust to the absence of clear resource typing standards or technical qualifications for drone pilots.

C: “Ultimately, the people that we trust to work with come from experience working with them. You go out on a limb and you try it, and then if they actually can do the things they say they can do, then that's about the only way [to gain trust]. But there's no resource typing available yet, no personnel qualifications in [using drones for] emergency management, emergency services.”

C: “I think that one of the things I am most wary about is that … drones are so much more accessible. There are a lot of individuals who have some experience with drones. What that leads to is sometimes an assumption of expertise that isn't quite as full as is needed.”

#### Drone user respondents expressed distrust of “disaster tourists,” whom they felt were unqualified to fly during a disaster

A majority of drone users expressed displeasure with people that they deemed “disaster tourists.” These users differentiated themselves (in the absence of formal mechanisms) by emphasizing prior experience and the ability to be “self-sufficient.” They categorized “disaster tourist” individuals into two groups: unaffiliated people who used their personal drones to take photographs or video often for social media posts and aspirant disaster response drone pilots who lacked knowledge of the regulatory and/or the disaster response environment.

A: “You understand going to a disaster that there's a lot of people [who] go into a disaster not knowing what they may see, not knowing how the incident command system works, how they integrate into that system. Those are challenges that people don't understand. They don't understand it. In most disasters, there's not amenities for them. So, they become a burden upon the responders, not directly but indirectly.”

B: “Don't go unless you're requested, and don't go unless there is an infrastructure in place that support[s] you, or you can bring with you. That's a huge issue. That's not really just UAVs, but we see a lot of that. A lot of people want to come help, and that's great, and they're not qualified. I don't mean from a technical standpoint. They don't have any idea what they're getting into.”

#### Drone users expressed frustration regarding poor communication, data sharing, and coordination between organizations involved in disaster response

All interviewees expressed some level of frustration with a lack of coordination and communication between different disaster response organizations that used drones or drone-collected data. Some directly pointed to a lack of a central coordinating body for disaster response drone users. One interviewee observed that there is no way to “talk to everybody” using drones in a disaster response, leading to problems of overlapping airspace use, duplication of data, and different standards for data collection.

A: “We’ve learned that the question to ask is “How do we get people to come together and work on the same sheet of music? You've got to have policies and procedures. You've got to have strict training standards. They've got to be able to communicate.”

B: “Honestly, the problem is, the community that might be doing this kind of work is very large, and we don't have a way to talk to everybody. You know, we can put the word out through as many methods as possible, and it's just not necessarily going to work each time.”

#### Drone users were motivated to prove that the technology is useful for specific disaster response purposes

Interviewees were largely already convinced of the usefulness of drone technology for damage assessment purposes. They hoped that their efforts during Harvey and Irma would provide evidence for the technology’s suitability for disaster response efforts, and help to make the case for integrating it further into disaster response systems. Some interviewees stressed that while they found UAVs to be useful in specific circumstances, they are not practical tools for all tasks. Respondents described themselves as still in the process of “figuring out” what these optimal use cases might be.

A: “We wanted to show the world that this can be done legitimately, legally, and safely, and still get accomplished without being roped off with the red tape at the FAA or local municipalities.”

B: “I know that in the areas that I worked in, [people] were amazed at how the technology was able to help them, they were excited…. The industry is growing by leaps and bounds and people are starting to learn what it can do for them, especially in case of an emergency.”

### Technical

Technical themes are concerned with UAV hardware and software as well as management of the UAV-collected data.

#### While technical challenges were reported during the observational stage of this study, respondents largely did not identify either hardware or software factors as significant obstacles to their work

During both the Florida and Texas observational stages of this study, drone pilots described a number of potential challenges around variables such as environmental factors (e.g., rain or wind) delayed UAV deployment, limited battery capacity, and challenging data transfer mechanisms. Our respondents were largely able to successfully plan around hardware limitations prior to deploying into the field, by methods such as estimating the number of batteries they might need to charge for a given days flight operations, pausing flight operations during windy or rainy conditions (which largely did not occur during both the Harvey and Irma response periods we observed), and keeping their UAVs well-maintained and ready-to-go prior to deployment. In the field, all UAV-using respondents that we observed relied upon checklists, maintenance records, and other techniques for ensuring that UAV platforms remained operational. Respondents reported that they were already familiar with the UAV flight planning and data-collection software that they used in the field, and that it largely operated as expected.

Only one respondent reported a truly significant so-called “tool to task” match issue in the field, pertaining to the use of a tethered, immobile UAV system for disaster response purposes. As the UAV was only able to view a limited area and was not capable of adding geo-tagged coordinates to the imagery it collected, it was ultimately not as useful for disaster responders as fully mobile, GPS-enabled UAV platforms were.

Beyond UAV platforms and UAV flight planning and data-collection software, some respondents reported challenges with transferring data from storage cards located on the drone to the intended data-end-users. UAVs are capable of collecting terabytes of imagery data during a disaster response deployment, and such large quantities of data are difficult to transfer electronically during disasters, when mobile data and internet connections may be unavailable or operating at unusually slow speeds. Some respondents attempted to upload UAV data to organizational web portals, but found this to be a challenging and often lengthy process. Respondents largely resorted to “handing-off” data utilizing portable storage drives to intended recipients in person as a result.

Overall, even these technical difficulties were not cited as significant obstacles to UAV operations by our respondents. Respondents were considerably more likely to cite non-technical challenges related to organization, communication, and the regulatory environment as key impediments to their use of UAV technology, than they were to identify impediments related to specifically technical issues.

#### Drone users saw themselves primarily as data collectors not data analysts

Almost all interviewees described themselves primarily as data collectors and did not view their role as data analysts or disaster response decision-makers. They viewed themselves as responsible for the security of the data while it was in their possession, but this responsibility transferred to their tasker when the data was “handed off”. One interviewee (quoted below) expressly described the avoidance of data analysis as a means of avoiding some ethical dilemmas.

A: So if we were to see something like [possibly illegal items], then it’s an ethics question [of] “Do we alert the authorities? Which then just makes things even worse. So that’s why we don’t do a lot of the analysis. We collect the data, we pass it off to whatever authority we are working with or whichever agency we are working with, and we let them deal with that. We are just there to help them get the information, and then they can decide where to go from there.”

B: “We don't release information that we collect. Just about everything we do in the field, we're doing for a partner agency, so that gets turned over to them. That's their data to release or do what with. And no, we don't have any policies regarding vulnerabilities on the aircraft.”

#### Drone users often are uncertain about what happens to the data after they collect it and frequently lack feedback from end-users

Almost all interviewees indicated that they lacked specific knowledge of how the data they collected was ultimately used by decision makers. There was general concern about the integration and utility of the data collected. Some respondents did describe receiving immediate feedback on the data they collected. They observed this was more likely to happen during the immediate disaster recovery stage, and in circumstances in which they captured images of an individual’s home. Interviewees generally expressed a preference for receiving feedback, but did not view this as an acute problem or as a disincentive to their work.

A: “There is probably a lot of duplication of effort going on, as well as probably a lot of imagery being collected that no one is looking at. This is effectively useless from a decision-making standpoint, because no one's looking at it …The biggest problem that we discovered after this hurricane season is, how do we streamline this effort and effectively mix satellite, high-altitude manned, low-altitude manned, and UAV operations so that we're not duplicating effort, and so that we're only providing products that people can use? Because right now, that's definitely not the case.”

B: “They [the recipients of the drone data] don't have data analysts or people to look at the data right when we collect it or right within a few hours or let's say a couple days of it. With them still fighting the fire, that data usually goes to the back burner and then they'll say oh, I forgot we had this data. Whereas in Fort Bend County, we had engaged stakeholders that were giving us feedback immediately.”

## Discussion

Overall, our outcomes reflect the challenges of any new technology entering the field of disaster response, ranging from matters related to public perception to the creation of effective incorporation and adequate credentialing mechanisms. Similar to the use of satellites, geographic information systems, and, historically, even cameras, there is considerable public caution regarding UAVs and privacy concerns.

Drone pilots who flew during Hurricanes Harvey and Irma—aware of these common, negative perceptions of the technology—emphasized transparency and community engagement in their post-disaster work. Their attention to these methods appears to have been effective. We observed that the public response to drone use during the hurricanes, both on social media and in traditional media coverage, was largely very positive. This is consistent with literature that evidences that the general public is more likely to accept UAVs when they perceive them as a technology that is being utilized by actors who are working to support their the community. [57,58]

Furthermore, when viewed through the lens of the “emergent response group” construct that explores relationships between emergent disaster response communities that are often united by novel technologies or methods, as discussed below, the challenges faced by those using UAVs or utilizing UAV-collected data in post-hurricane response are not only comparable to these earlier use-cases, but are also nearly expected. [59] Practice often precedes a systemic understanding of how new technologies and the individuals who use them ought to be incorporated into existing organizations and systems. In the absence of standardized methods and practices for using a new technology, it is very difficult to develop regulations, training programs, and widely-agreed upon professional credentialing systems. Viewed through this lens, our findings are not only complementary to historical trends, but also to existing literature evaluating the contemporary use of UAVs for international disaster response.

### Drone pilots are aware of potentially negative perceptions of drone use by the public and the need to elicit community support for their actions. However, the practice of transparency, face-to-face interactions with residents, and the inclusion of community representatives have strengthened the perception of UAVs as being used for “public good”, and likely lead to a predominantly positive community response

The drone pilots we spoke with were keenly aware of the possibility that their drones could be perceived negatively by the communities in which they operated. Some were “surprised” by their largely positive interactions with the community, in light of recent examples of civilian drones being shot down by property owners in the United States. For both ethical and practical reasons, drone pilots felt they needed to negotiate access to disaster-affected areas, both with community leaders and with local citizens and property owners. We observed that interviewees with more experience using drones for disaster response were more aware of the importance of personalized community interactions, were more likely to have considered the impact of public privacy concerns, and were more likely to have constructed their own personal or small-group standards regarding community engagement and data sharing prior to deployment.

Explanations for this positive reception from the community are three-fold: drones perceived as being used for the “public good”, the incorporation of face-to-face interactions with community members, and the inclusion of county or community representatives in flight teams. Some respondents opined that the positive response was because community members found clear value in the data they collected, thereby fostering public good will towards the pilots and a softening of attitudes around UAVs.

Recent public opinion research indicates that drone use for “public good” purposes, such as disaster response, is more popular than is commonly assumed. Sakiyama et al. in a 2016 national online survey found 94% support for the use of drones by domestic police or search and rescue activities, in comparison to 47% support for police use of drones for crowd management [57]. A 2017 public opinion survey found especially strong public support for drone use for firefighting, search and rescue, and conservation, while respondents were more likely to oppose drone use for real estate, business, and hobby purposes [58]. Other researchers have found that public support for drone use was predicted primarily by the drone’s perceived purpose, and not by message framing or by the UAV end-user, themselves [60].

The face-to-face interactions observed and described by interviewees likely led to the communities’ perception that drone operations were for “public good” purposes. One interviewee emphasized the importance of “transparency” in community engagement work. To put this into practice, his team would “answer questions, talk to people, show them what they’re doing”. During the case study, researchers observed a neighborhood resident request that the UAV team photograph her house, which they willingly did. While a respondent noted that these interactions can provide “closure for the community” or permit people to see if they “still had a house”, the availability and transparency of the drone pilots likely reduced community members’ perception of threatened privacy. A recent Danish research project found that community members’ perception of privacy disturbance from drones was linked to their ability to gauge who was flying it, and for what purpose.[61]

The small drones used by Harvey and Irma responders have short operating ranges, often less than a mile. Furthermore, the FAA mandates that small UAVs must remain “within visual line of sight” of the pilot in most circumstances. These two factors meant that the UAV pilots interviewed for this study were always within close physical proximity of their drones. Concerned or curious community members were generally able to physically locate and approach the drone pilots. The drone pilots, for their part, largely expected these interactions, and were prepared to answer questions and to offer explanations of their activities.

Lastly, the intentional inclusion of county or community representatives into teams conducting UAV flights was cited as another positive influence on public perception. One interviewee reported that a county representative accompanied their UAV teams on a daily basis as missions were conducted in and around Houston. This individual was able to answer community members’ questions and direct them to resources, enabling the team to engage meaningfully with the community without hindering their focus on operating the UAV.

Another respondent noted that their organization sent non-drone-operating volunteers with extant community partnerships into the field alongside its drone damage assessment teams. Similarly, these individuals were tasked with engaging with residents and answering questions. The presence of community-linked personnel feasibly provided a touch-stone that increased trust, provided valuable information about resources, and lead to the support of UAV operations as a “public good.”

### The existing disaster response and regulatory system is being challenged by the rise of drone-using “emergent” response groups and individuals. Identifying the utility of UAV-derived data and the particular characteristics of these “emergent” volunteers is critical for effective incorporation of the technology

Some of the volunteer drone pilots observed during Harvey and Irma might be classified as “emergent response groups.” Majchrazk defines these as “groups with no preexisting structures such as group membership, tasks, roles, or expertise that can be specified ex ante.”[59] They are “characterized by a sense of great urgency and high levels of interdependence, operating in environments that are constantly changing as new information arrives about needs for victims and resources.” [62] They commonly adopt a“learn by doing” problem-solving model, reliant on improvisation and intuition.

These definitions accurately describe one of the volunteer drone organizations observed in the field, which originated from a spontaneous decision among members of a UAV hobbyist Facebook group to offer drone services to those affected by Hurricane Harvey. While this organization was hastily created and remained informal during its existence in Harvey, group members attempted to integrate themselves into the disaster response system and to adhere to FAA regulations. These emergent response group members saw themselves as filling a void left by other actors, convinced that the UAV-derived data they could collect would directly benefit disaster-affected communities. When they perceived themselves as obstructed by the FAA or more established disaster response organizations, they complied with regulations, but opined that these obstacles provided a disservice not just to themselves but to community members, who they believed they could imminently help.

Respondents perceived a difference between organizations that attempted to integrate themselves into existing disaster response systems and “outsider” drone pilots.[62] These “outsiders” did not appear to coordinate flight missions with other organizations and did not appear to adhere to FAA regulations. They were either not aware of or chose not to integrate themselves with the disaster response system. All interviewees were generally critical of these “outsider” drone pilots, some labeling them as “disaster tourists”. They described these activities as a danger to their own continued access to and use of the technology, and a danger to other disaster responders and the reputation of the UAV industry. This aligns with how informal disaster response volunteers are often generally viewed by disaster responders who work within the system and who view themselves as more professional–as problematic, potential nuisances.[63]

Still, most interviewees acknowledged that these drone pilots could be helpful to disaster responders, and cited instances from Harvey and Irma in which the drone imagery they uploaded to social media was used for disaster assessment and search and rescue operations. Some interviewees felt that these flights were essentially inevitable in the absence of FAA enforcement, but also noted that attempting to convince “outsider” pilots to work within the system might be a more pragmatic solution than punishing them. This assessment is shared by a number of disaster researchers, who agree that this “emergent” volunteer behavior, or the “convergence” of non-professional responders towards a disaster response area is inevitable.[64] Stallings and Quarantelli note that this is in part because citizens will inevitably identify needs during disasters that organized disaster response groups and agencies are not aware of or cannot address. [65]

Those respondents who had considerable prior experience with UAV technology but less prior disaster response experience expressed particular frustration with what they perceived to be the “slow” and “bureaucratic” nature of large disaster response and regulatory organizations. We observed similar conversations critical of this aspect of the disaster response system on social media networks. Interviewees also wondered if this so-called “red tape” from the FAA might be one reason why some altruistically-minded UAV users were choosing not to engage with the disaster response system.

The commercial drone industry and the drone enthusiast subculture that has developed around it has historically had a complex and occasionally contentious relationship with the FAA. We observed that drone users during disaster who hewed closer to the definition of “informal” volunteers or “emergent group members” were more likely to express this frustration. However, interviewees from both groups with prior disaster response and from those with less experience often cited the FAA’s perceived inability or unwillingness to enforce restricted air space from UAV-pilots without authorization as problematic, and linked it to a sense of feeling unprotected and undervalued.

Both airspace regulators like the FAA and existing disaster response organizations are faced with an overarching problem: how should emergent response groups who use UAVs be integrated into existing disaster response structures and procedures? One interviewee observed that these emergent volunteers and groups are “not being tied into an organized network,” and noted his particular concern with the lack of a “command center” model commonly used in public safety.

This is not a new conundrum. Whittaker et al. observe that the established disaster response system often does view “emergent” volunteers as “a nuisance or liability,” and often does “undervalue” their efforts, making effective integration difficult.[66] Thus, it remains uncertain exactly how UAS technology and personnel ought to best be incorporated into emergency command centers. Answering that question will require considerably more inquiry into the utility of UAV-derived data and the particular characteristics and makeup of “emergent response groups” and “outsider” drone pilots.

### Because there is little formal organization of drone-based disaster response, organizations providing UAV services lack clear means of assessing the credibility of other drone users’ knowledge and expertise. Instead, they rely upon a trust-based system linked to existing personal relationships to source UAV pilots

Literature in transactional memory theory [67] describes credibility in the knowledge of other members as an essential element of coordinated efforts, which is in keeping with observations of our study. [68] Majchrzak et al. specifically note that “emergent response groups” are particularly challenged by the problem of validating the expertise of other group members, especially under volatile, challenging conditions. [59]

The UAV pilots interviewed described a clear dilemma related to assessing credibility in member knowledge. In the absence of any clear or formalized means for doing so, they had to rely upon personal relationships and history. One newly-established “emergent” volunteer organization observed was composed of members who had all previously interacted with one another on a Facebook group, but had not actually met in “real life” prior to deployment. Although they had not physically met some of their teammates, they still found their knowledge to be credible, based on prior interactions. Another interviewee with a longer-established disaster response organization noted that he had also found social media “extremely useful” for identifying and recruiting qualified UAV pilots.

Formal certification and testing mechanisms are one way in which individuals can assess the knowledge and expertise of others. However, methods for objectively assessing the skill of small drone pilots remain limited. In 2017, FEMA released resource typing definitions for UAV teams that mandate UAV users complete FEMA-offered trainings on the overall disaster response system, in addition to holding a FAA remote pilot license. [69] The FAA’s test for commercial UAS pilots is an entirely written examination that does not test practical skills, nor does it include any specific information geared towards disaster response. Most interviewees stated that they found it to be inadequate for their purposes, and expressed a preference for the development of a hands-on, practical test for assessing a pilot’s UAV piloting skills. While a number of companies and educational institutions now offer training and certifications for UAV use in disaster response, there is no standardized, national training or accreditation body. [70,71]

Additionally, there is no single, widely-acknowledged public, private, or government organization for disaster response UAV users in the United States. No organization exists analogous to the National Association for Search and Rescue, which offers training, exams, and certification. [72]A number of relatively informal, not-for-profit organizations like the “S.W.A.R.M Drones Network” operate via websites and Facebook groups. [73] These groups allow individuals to be listed on their rosters and share information about scenarios in which UAV volunteers may be wanted, but do not offer skill assessment methodologies or certifications. Some companies and organizations attempted to function as informal mechanisms for organizing and tasking UAV pilots during the Harvey and Irma responses. However, there are concerns regarding the ability of these bodies to assess member expertise, their lack of communication with other responders, and the accuracy of mission reporting.

The current lack of a widely acknowledged body for disaster response UAV users and the absence of a standardized accreditation mechanism for UAV pilots operating in post-disaster environments is a critical gap that needs to be addressed. In the absence of such a body, personal interactions will continue to be a part of sourcing skilled UAV pilots despite the fact that it fosters perceptions of inadequacy and nepotism in the field of UAV technology for disaster response.

### Coordination challenges and a lack of agreed-upon codes of conduct or methods for skill-assessment are not unique to the U.S. setting

Participants who used UAVs during the response to the 2015 Nepal earthquakes have described very similar coordination challenges to those described by our participants in the U.S. [74]While drone use is becoming more commonplace in international humanitarian response, it is still unclear where UAVs fit into the overall humanitarian cluster system. There is no centralized system for tasking, compiling, and sharing humanitarian UAV data across aid agencies or NGOs. [75]The Humanitarian UAV Network, launched in 2014, functions as a central hub for individuals and organizations who use UAVs in disaster response: the group claims to have over 3,200 members across over 120 countries. [76] The Humanitarian UAV Network’s Code of Conduct is one example of an international effort at creating non-governmental best practices for UAV use during disaster. However, these best practices do not appear to be widely known or used in the United States, and they have not yet been formally adopted by any large aid organizations. Many non-U.S. humanitarian researchers and practitioners describe similar problems as those reported by our interviewees related to interpreting disaster response data and measuring the impact that this data had on their decision making process. [77]

## Limitations

The sample size of ten interviewees is small and unevenly distributed between drone-deployers and end-users of UAV-collected data. This is in part due to the small population of people who used UAVs or UAV-collected data during the responses to Hurricanes Harvey and Irma. It is also due to the considerable organizational and communication challenges inherent to the UAV ecosystem, which limited the researchers’ ability to identify, contact and gain the consent of new respondents. While this sample size is small, the research team does not believe that it represents a major shortcoming in these results. The goal of attaining an acceptable degree of data saturation as described by qualitative experts was achieved. [78–80] This study had a particularly narrow focus, and the experiences and opinions explored are intrinsically rare in the available universe of interviewees.

We also defend our limited sample size on the basis of data triangulation as described as Denzin et al. [81] The qualitative case-study approach incorporated many other sources of information beyond semi-structured interviews, including social media postings, direct field observation, news and media reports, and other academic literature. There is considerable corroboration between this study’s findings from the coded interviews and these additional sources.

Perhaps due to the sensitive nature of disaster-response decision-making, or simply time availability, end-users of UAV-collected data are underrepresented. Further respondent imbalances should be explicated, with the cross-case analysis featuring considerably fewer respondents involved in post-Irma disaster management in comparison to post-Harvey efforts. There are also conspicuously few female-identified respondents. While these disproportions are likely reflective of the ecosystem, itself, we acknowledge that it results in outcomes biased towards the perspectives of UAV-pilots, those involved in Hurricane Harvey response, and male-identified individuals.

Finally, our own identities as researchers are relevant to the outcome of this study. One researcher has conducted research and published popular writing on civilian UAVs for a number of years, was known to others in the relatively small circle of UAV professionals prior to this work, and has previously interacted with two of the study participants. These prior interactions facilitated respondent identification and field observations, but may have biased research subject interactions. Both researchers self-identified as academic observers both in the field and during interviews. Identification as such prejudiced subjects in both amount and content of disclosure of perspectives, depending upon a priori illicit bias.

## Recommendations and future study

This case study is exploratory in nature and is intended to serve as a launch point for further research into the use of UAVs for damage assessment purposes during disaster. Our findings indicate the need for further study regarding the organizational structure of UAV use in disaster response, community perceptions of UAV use, and how UAV pilots in disaster fit into existing disaster theory and research.

There is a considerable body of disaster studies research that focuses on how new technologies and voluntary, “emergent” groups are integrated into existing disaster response systems. However, there is little existing research that attempts to apply these well-developed models to the relatively new use of civilian UAVs in disaster response. Little is understood about the profile or characteristics of UAV users in disaster response. A better understanding of these demographics will aid the development of systems for coordinating efforts and determining the value of UAV-collected data. Methodologies developed in this study for surveying U.S.-based, UAV disaster responders could be applicable to conducting such research with non-U.S., UAV disaster responders.

Based on our organizational observations, we suggest disaster responders consider what will be required to standardize and formalize drone use in disaster. We observed both volunteer and paid drone users conducting damage assessment work during Harvey and Irma, and it remains unclear if a volunteer model or one in which damage assessment work done with drones is conducted by paid contractors is preferable. Either model should be supported by the creation of a national organization dedicated to UAV use during disaster response, similar to the National Association for Search and Rescue. Such an organization might be able to fill the observed gap in standardized skills testing and training systems designed specifically for drones.

The “data black box” that our interviewees described separates UAV users from the ultimate outcomes of the data that they collect. There is still little known about the actual impact of UAV data collection on disaster response outcomes. Future research should attempt to describe how the presence of UAV pilots and the data that they collect has altered disaster response operations.

Our research also indicates that little is known about how the U.S. or non-U.S. public specifically views the use of drone technology in disaster response efforts. It is often assumed that the public distrusts and dislikes drones. There is a need for survey research that addresses public perceptions of drones in disaster response, including demographic factors such as the identity of the operator, geography, economic status, and racial and gender identities.

## Conclusion

In this research, we used an exploratory case study methodology to investigate the actions of UAV users during the 2017 Harvey and Irma hurricanes in the United States. We found that the UAV ecosystem consisted of primary deployers of UAVs, primary utilizers of UAV-captured data, and actors that both deployed drones and utilized self-captured data. There appeared to be few individuals operational in the response who were equally conversant in both UAV operation and in the disaster response system. We described the regulatory environment acting upon UAV users in disaster response and the absence of disaster-specific standards related to drone use and privacy protection in the U.S.

From the results of the semi-directed interviews conducted, we conclude that in order for UAVs to be effectively and ethically incorporated into disaster response systems, four thematic domains need to be addressed, including ethical, regulatory, programmatic, and technical considerations. Drone users were met with a positive response by community members, but this likely resulted from practices that included transparency regarding data use, face-to-face interactions with residents, and the inclusion of community representatives in flight teams. Interviewees expressed dissatisfaction with the FAA’s inability to enforce controlled airspace rules and the lack of disaster-specific regulations. They also found the FAA’s written test to be inadequate for assessing pilots’ skills in the disaster context and relied on personal relationships to guide UAV pilot hiring in lieu of formalized evaluation mechanisms.

Overall, there was a lack of coordination and communication among UAV users and between UAV users and other organizations utilizing UAV-collected data. Without a consensus on the utility of UAV-collected data in disaster response decision-making or an understanding of best practices, organizations and personnel create their own methods regarding data collection, management, security and sharing. When compared to previous research on “emergent response groups”, these observations are analogous to those faced by other communities utilizing novel technologies or methods that arise in the disaster management space. And these challenges are not unique to the U.S. context, with the use of drones for disaster response in international settings leading to similar conundrums and discourse. Further study regarding the utility, best practices, regulations and organizational structures needed for effective and ethical UAV utilization in disaster management is exigent.

## Supporting information

S1 File Consolidated criteria for UAV use in damage assessment. Completed consolidated criteria for qualitative research form.
(DOCX)

S2 File Semi-directed interview guide. Semi-directed interview questions used during post-hoc interviews.
(DOCX)

S3 File Data extraction tool harvey. Data extraction tool used to collect social media and news postings about UAV use during Hurricane Harvey.
(XLSX)
