# Supplementary material for: Flying into the hurricane: A case study of UAV use in damage assessment during the 2017 hurricanes in Texas and Florida
Source: PLoS One. 2020 Feb 5;15(2):e0227808. doi: 10.1371/journal.pone.0227808 (PMC7001970; doi:10.1371/journal.pone.0227808)
Supplement: S2 File — Semi-directed interview questions used during post-hoc interviews. (DOCX) [file pone.0227808.s002.docx]

**(Post-Hoc) Semi-Directed Interview Questions for “Flying into the hurricane: A case study of UAV use in damage assessment during the 2017 hurricanes in Texas and Florida.”**

**Domains of Inquiry:**

**General Use:**

1. Are you using remote sensing data in your disaster response efforts?
2. If so, what kind?
3. From where is your data coming?
4. How do you vet your data source?
5. Do you or your team have previous experience using remote sensing data?
6. From your perspective, how have RS (UAVs and satellites) changed disaster response?

**Ethics/Legal:**

1. FAA regulations and enforcement
   1. What is your understanding of the FAA regulations surrounding UAV use?
   2. Do you have an organizational policy regarding legal UAV use?
   3. Have you had experiences with the FAA and/or other regulatory bodies regarding UAV use?
2. Perceptions of the affected
   1. What are the affected communities’ perceptions of your work?
   2. What are you hearing regarding the communities’ response to UAVs?
   3. Are there any ethical concerns on your part about using UAVs?
3. UAVs and Disaster Response Fraud
   1. Have you heard of any instances of UAVs being utilized in fraudulent fundraising campaigns?
   2. Are there any other overtly unscrupulous incidences regarding UAVs that you’d like to share?

**Operations:**

1. Personnel/volunteers
   1. How have you sourced UAS professionals?
   2. How have you credentialed your UAS professionals?
   3. Did you find this process challenging in any way?
   4. How would you change it?
   5. From your perspective, is there a role for a special certification or professionalization process?
2. Organization-associated UAVs
   1. Have you experienced any specific limitations to using your own UAVs?
   2. Can you identify/distinguish others’ UAV? How do you visually/digitally distinguish your own craft?
   3. How do you decide where to fly? Do you take into account other RS or needs assessment strategies?
3. Data strategy: collection, storage, analysis, dissemination
   1. What are your methods of data accumulation, cleaning and storage?
   2. Who and how are you analyzing your data?
   3. How do you incorporate other data sources for triangulation?
   4. Are these data filling a gap?
4. Data to operations strategy
   1. How are these data informing your response operations? (e.g?)
   2. To whom and how are you disseminating these data?
   3. What is your turnaround?
   4. How do you M&E your RS operations?

**Social media and hobbyist drones:**

1. Experience w volunteer drone force
   1. What, if any, is your experience with UAV pilots wanting to volunteer?
2. Interaction w independent hobbyist drones
   1. What, if any is your experience with independent hobbyist drones?
   2. What is your perspective on independent hobbyist UAV use for civilian journalism?
3. Utilization of social media RS data
   1. What, if any, experience have you had with RS data sourced from social media?
   2. How does these data become incorporated into your operations?
   3. Do you/your organization disseminate RS data via social media?

**Survey Questions:**

1. Is your organization currently using information collected by satellites in your decision-making process? (Y/N)
2. If you answered “yes,”how are you using this information? Check all that apply. (Determining which areas should be prioritized by responders, getting baseline information on the area, communicating with the public, communicating with the media, gathering information for later analysis).
3. Where are you getting satellite information? Check all that apply: (NOAA, FEMA, DOD, Digital Globe or other commercial providers, Google Earth, social media sources, Other….)
4. Is your organization currently using information collected by drones in your decision-making process? (Y/N)
5. If you answered “yes,”how are you using this information? Check all that apply. (Determining which areas should be prioritized by responders, getting baseline information on the area, communicating with the public, communicating with the media, gathering information for later analysis).
6. Where are you getting drone-collected information? Check all that apply: (Volunteer drone pilots collecting information at your request, volunteer drone pilots who are sharing information you did not request with you, drone pilots employed by your organization, drone imagery posted to social media, Other).
7. Have you used satellite imagery to inform your response before? (Y/N)
8. Have you used drone imagery to inform your response before? (Y/N)
9. Do you have specific ethical concerns about using imagery from satellites and drones in your response? If so, check all that apply: (Risk to personal privacy, risk to personal privacy, risk of information being used by other actors if it is made publicly available, risk to personal safety of individuals on ground from drones, risk to disaster responders, Other (write-in?), I do not have specific ethical concerns.
